# A Blended Learning Course on the Diagnostics of Mental Disorders: Multicenter Cluster Randomized Noninferiority Trial

**DOI:** 10.2196/54176

**Published:** 2024-11-27

**Authors:** Gabriel Bonnin, Svea Kröber, Silvia Schneider, Jürgen Margraf, Verena Pflug, Alexander L Gerlach, Timo Slotta, Hanna Christiansen, Björn Albrecht, Mira-Lynn Chavanon, Gerrit Hirschfeld, Tina In-Albon, Meinald T Thielsch, Ruth von Brachel

**Affiliations:** 1 Mental Health Research and Treatment Center (FBZ) Faculty of Psychology Ruhr University Bochum Bochum Germany; 2 German Center for Mental Health (DZPG) Bochum-Marburg Germany; 3 Clinical Psychology and Psychotherapy Department of Psychology University of Cologne Cologne Germany; 4 Clinical Child and Adolescent Psychology Department of Psychology Philipps University Marburg Marburg Germany; 5 Faculty of Business University of Applied Sciences Bielefeld Bielefeld Germany; 6 Clinical Child and Adolescent Psychology and Psychotherapy Department of Psychology University of Mannheim Mannheim Germany; 7 Work and Environmental Psychology Department of Psychology University of Wuppertal Wuppertal Germany

**Keywords:** diagnosis, structured clinical interviews, blended learning, dissemination, therapist training, clinical interview, clinical diagnosis, clinical practice, psychology students, diagnostic test, health personnel, mental health services, mental health

## Abstract

**Background:**

Clinical diagnoses determine if and how therapists treat their patients. As misdiagnoses can have severe adverse effects, disseminating evidence-based diagnostic skills into clinical practice is highly important.

**Objective:**

This study aimed to develop and evaluate a blended learning course in a multicenter cluster randomized controlled trial.

**Methods:**

Undergraduate psychology students (N=350) enrolled in 18 university courses at 3 universities. The courses were randomly assigned to blended learning or traditional synchronous teaching. The primary outcome was the participants’ performances in a clinical diagnostic interview after the courses. The secondary outcomes were diagnostic knowledge and participants’ reactions to the courses. All outcomes were analyzed on the individual participant level using noninferiority testing.

**Results:**

Compared with the synchronous course (74.6% pass rate), participation in the blended learning course (89% pass rate) increased the likelihood of successfully passing the behavioral test (odds ratio 2.77, 95% CI 1.55-5.13), indicating not only noninferiority but superiority of the blended learning course. Furthermore, superiority of the blended learning over the synchronous course could be found regarding diagnostic knowledge (β=.13, 95% CI 0.01-0.26), course clarity (β=.40, 95% CI 0.27-0.53), course structure (β=.18, 95% CI 0.04-0.32), and informativeness (β=.19, 95% CI 0.06-0.32).

**Conclusions:**

Blended learning can help to improve the diagnostic skills and knowledge of (future) clinicians and thus make an important contribution to improving mental health care.

**Trial Registration:**

ClinicalTrials.gov NCT05294094; https://clinicaltrials.gov/study/NCT05294094

## Introduction

The reliable and valid diagnosis of mental disorders is associated with a more favorable therapeutic course and outcome [1,2]. However, although structured clinical interviews are acknowledged as the gold standard for diagnosing mental disorders [3-5], clinicians often rely on unstructured, experience-based explorations of symptoms [6,7]. As a result, both the under- and overdiagnosis of mental disorders are common [8-10], leading to undertreatment [11] or inappropriate or unnecessary psychotherapy or medication [12-14]. In view of the high rates of misdiagnoses, there is an urgent need to improve diagnostics of mental disorders by disseminating evidence-based assessment procedures into clinical practice. While there is increasing awareness of the importance of disseminating evidence-based treatment [15,16], the foundation of successful treatment, namely evidence-based diagnostics, has not been sufficiently addressed in dissemination research [17,18]. Therefore, the aim of this study was to develop and evaluate a blended learning course to disseminate evidence-based diagnostics of mental disorders.

Learning environments can be classified into 4 groups based on their modality (offline vs online) and synchronicity (synchronous vs asynchronous). Traditional classroom teaching combines synchronous with offline teaching, while webinars are an example of synchronous online teaching. Asynchronous teaching can be realized online through learning management systems or offline with the use of printed learning materials. Recent meta-analytical evidence indicates that there are no significant differences in learning outcomes between synchronous offline and online learning [19,20], as well as asynchronous online instruction [20]. In addition, there are various combinations of these learning environments, including blended learning, which combines (synchronous) offline and (asynchronous) online learning [21]. This approach addresses the limitations of both traditional offline and online learning, such as reduced student engagement and inconvenient time and space requirements [22]. By combining the best of both approaches, blended learning provides a more effective learning experience. Recent meta-analytical evidence suggests that blended learning is consistently more effective than either synchronous online or offline learning alone and is better accepted than traditional offline teaching [22-24].

In addition, a blended learning approach may be especially appropriate for disseminating evidence-based diagnostics, as it addresses challenges commonly associated with its training and dissemination in a face-to-face setting [25]. First, time and costs that are required for training in evidence-based methods act as important barriers for attendance [26]. Web-based or blended training methods can overcome this barrier by allowing clinical knowledge and skills to be trained in a time- and cost-efficient, easily accessible, flexible, and highly standardized way [27-29]. Second, viewing case studies of and practicing diagnostic situations are essential for acquiring diagnostic skills. As videos of diagnostic situations with simulated patients can be included in the asynchronous online part, whereas practicing in role plays can occur during the synchronous face-to-face part of the course, a blended learning approach allows learning practical diagnostic skills in a flexible and time-saving way. Third, clinicians underestimate patient acceptance of structured interviews and seem to have various preconceptions against their use [6]. These preconceptions can be reduced by addressing them explicitly and by intensifying training in the implementation of structured interviews [18,26,30]. By conveying content in a highly accessible and standardized way, blended learning courses can contribute to the intensification and standardization of training in structured diagnostic interviews and hereby reduce prejudices against their use.

Although the use and acceptance of online teaching methods increased globally during the COVID-19 pandemic [31,32], until today, only a few studies evaluated blended learning in randomized controlled trials [33-36]. Despite the high relevance of disseminating evidence-based diagnostics into clinical practice, to our knowledge, blended learning for teaching diagnostic skills was not yet evaluated at all. We aimed to fill this gap by conducting a cluster randomized controlled trial at 3 German universities and comparing a blended learning course with regular face-to-face teaching in a noninferiority analysis at the individual participant level. As there is evidence that the impact of training on more experienced practitioners does not last over time [37,38], and it is considered that such training may be more effective for those at the beginning of their clinical careers [39], we targeted a relatively inexperienced sample of preprofessionals, specifically undergraduate psychology students.

We hypothesize that students’ interviewing skills, knowledge acquisition, and reactions in a novel blended learning course will be noninferior to those in traditional synchronous courses.

## Methods

### Study Design

The study was a multicenter cluster randomized controlled trial, comparing 2 university teaching formats: a blended learning course and a traditional synchronous course. A cluster randomization of courses was chosen because individual randomization of participants was not feasible given the constraints of the existing university setting. Clusters were 18 courses in clinical diagnostics at the 3 cooperating universities. Courses were randomly assigned to 1 of the 2 teaching conditions, stratified by study site. Participants could choose between courses in the online registration systems of the respective universities. To minimize any selection bias, course information available to participants (eg, content and instructor) was held constant in both conditions. Importantly, participants had no information about whether the teaching condition was synchronous or blended. Since the study was conducted at 3 different universities with different numbers of students and teachers, the number and size of courses at each center varied. There were 3 assessments—before the start of the courses (t1), before the last course session (t2), and after the last course session (t3).

While both teachers and participants were aware of the teaching condition to which they were assigned, key aspects of the assessment process were conducted under blinded conditions. Specifically, the actors portraying patients in the diagnostic skills test were unaware of the participants’ assigned teaching conditions to ensure impartiality in their interactions. In addition, the outcome assessors responsible for scoring the videotaped simulated diagnostic scenarios were also blinded to the participants’ group assignments, ensuring that the assessment of diagnostic skills was not influenced by knowledge of the training method.

The trial is registered on ClinicalTrials.gov (NCT05294094). Due to governmental protective restrictions in Germany related to the COVID-19 pandemic, synchronous sessions that were originally planned as face-to-face classes had to be conducted as online webinars. This deviation from the registered study protocol is addressed in the *Discussion* section of this paper, where the potential consequences and limitations are analyzed in detail.

In accordance with the journal guidelines for reporting randomized controlled trials of eHealth interventions, the CONSORT-EHEALTH checklist is included in Multimedia Appendix 1.

### Participants

A total of 3 universities took part in the study (Ruhr University Bochum, University of Cologne, and Philipps University Marburg). The eligibility criterion for clusters was an undergraduate course on clinical diagnostics at cooperating universities. Eligibility criteria for individual participants were (1) age >18 years, (2) undergraduate psychology students at a cooperating university, and (3) willingness to give informed consent online.

Participation in a course on the diagnostics of mental disorders was mandatory in the curriculum of the undergraduate psychology program at all cooperating universities. In total, 18 courses were offered, 10 of which focused on the diagnostics of mental disorders in adulthood and 8 of which focused on the diagnostics of mental disorders in childhood and adolescence. Participants were recruited over the course of 2 semesters between April 2021 and February 2022. During this period, the courses were attended by 400 students. Participation was possible for all students at each of the 3 measurement time points separately.

### Procedure

#### Overview

Before the start of the course, written informed consent was obtained. Study participation was voluntary and compensated with a test participant certificate (mandatory part of the study program) and a shopping voucher (€10-20 [US $10.84-21.68], value depending on the scope of study participation). While the synchronous online classes were held weekly from the beginning, the blended learning course started 6 weeks into the current semester for organizational reasons. The asynchronous online part of the blended learning course was accessible to all students through the Moodle learning platform.

#### Experimental Condition: Blended Learning Course

##### Overview

The blended learning course followed a flipped classroom model, in which asynchronous online lessons focused on content delivery and synchronous online sessions were used to apply and deepen clinical skills under the guidance of an instructor [40]. The course consisted of 8 asynchronous online lessons and 3 synchronous online sessions and was designed considering the current knowledge regarding the conditions under which blended learning is effective (eg, including case studies, interactive elements with personalized feedback, or collaborative activities during synchronous sessions [32,41]) and well accepted by students (eg, user-friendly and functional design [42]).

A detailed overview of the course content is illustrated in Table 1. Access to the asynchronous online course can be provided by the corresponding author (GB) on request.

**Table 1:** Structure and content of the blended learning course.

Part and lesson			Adulthood		Childhood and adolescence
**Part I: Asynchronous lessons**					
	Lessons 1-3: Diagnostic fundamentals and evidence-based assessment	1. Introduction to classificatory diagnostics, diagnostic approaches, and classification systems 2. The diagnostic process, standardized clinical assessment, and biasing influences on the diagnostic process 3. Structure, conduction, and development of the (Kinder-)DIPS-OA^a^		1-3. Same as adulthood	
	Lessons 4-7: Diagnostic criteria and conduction of the (Kinder-)DIPS-OA for specific disorders	4. Panic disorder, agoraphobia, social anxiety disorder, and generalized anxiety disorder 5. Bipolar disorders, major depression, persistent depressive disorder, and OCD^c^ 6. PTSD^d^, somatic symptoms disorder, and illness anxiety disorder 7. Anorexia nervosa, bulimia nervosa, and alcohol use disorder		4. ADHD^b^, oppositional defiant disorder, and conduct disorder 5. Separation anxiety disorder, specific phobia, and social anxiety disorder 6. Generalized anxiety disorder, selective mutism, and major depression 7. PTSD, OCD, and anorexia nervosa	
	Lesson 8: Evaluation of the (Kinder-)DIPS-OA	8. Evaluation of the (Kinder-)DIPS-OA, feedback of a diagnosis, difficult situations conducting the (Kinder-)DIPS-OA, and acceptance and psychometric properties of the (Kinder-)DIPS-OA		8. Same as adulthood	
**Part II: Synchronous Sessions**					
	Lessons 9-10	9. Apply skills and conduct the (Kinder-)DIPS-OA as the interviewer and as a patient with fellow students. Get direct feedback from peers and teacher. 10. Other nonspecified content was based on the students’ questions and interests (eg, questions regarding the diagnostic criteria, the diagnostic process, and how to conduct the [Kinder-]DIPS).		9-10. Same as adulthood	

^a^(Kinder-)DIPS-OA: Diagnostic Interview for Mental Disorders (in Children and Adolescents) – Open Access.

^b^ADHD: attention-deficit/hyperactivity disorder.

^c^OCD: obsessive compulsive disorder.

^d^PTSD: posttraumatic stress disorder.

##### Blended Learning Course—Asynchronous Lessons

In total, 2 separate versions of the asynchronous online course were developed—a version with a focus on the diagnostics of mental disorders in childhood and adolescence and a version with a focus on the diagnostics of mental disorders in adulthood. Both versions were parallel in content, except for age-specific diagnostic procedures and some of the disorders presented, as they typically occur at different developmental stages (Table 1). Furthermore, both versions focused on teaching the conduction of a semistructured diagnostic interview, the Diagnostic Interview for Mental Disorders – Open Access 1.2 (DIPS-OA1.2) [43], and the Diagnostic Interview for Mental Disorders in Children and Adolescents – Open Access (Kinder-DIPS-OA) [44].

Content, usability, and design of the online courses were formatively evaluated during development by students and research associates from the Ruhr University Bochum and the University of Koblenz-Landau.

Each lesson included an introduction and conclusion sequence, a downloadable handout, and final evaluation questions. In the disorder-specific lessons (4-7), video-based case studies (played by actors) were presented to illustrate the conduction of a structured interview and to allow participants to test and apply their acquired knowledge through interactive elements (eg, multiple choice questions, automatic feedback, and matching tasks). Participants were able to navigate through the lessons and subchapters independently; however, working through the course content in sequential order was recommended. A tutorial video was provided, explaining how to navigate through the course, as well as how to use the various interactive course elements. In addition, the course included a forum where participants could ask questions about the course content, which were answered by the first 2 study authors (GB and SK).

##### Blended Learning Course—Synchronous Sessions

Following the asynchronous online course, participants of the experimental condition took part in 3 weekly synchronous online sessions (90 min each). In these sessions, they could discuss questions about the asynchronous course content with a lecturer and apply their skills in role plays with the other participants.

#### Control Condition: Synchronous University Course

The synchronous university course took place in attendance and consisted of 11 weekly online sessions (90 min each), representing the usual teaching of clinical diagnostic knowledge and skills at the 3 cooperating universities. The teachers were instructed to work through mandatory content, which was based on the asynchronous online course to ensure comparability between the 2 conditions. Before the start of the course, a training session was held for the teachers. In addition, course material was provided in the form of Microsoft PowerPoint slides. In addition to the mandatory content, teachers were allowed to provide additional information relevant to the field of clinical diagnostics.

### Measures and Assessments

#### Primary Outcome: Practical Diagnostic Skills

The primary outcome was the students’ performance in a simulated structured diagnostic interview. At t2, course participants individually conducted a 15-minute section of a structured clinical interview (Diagnostic Interview for Mental Disorders [in Children and Adolescents] - Open Access; [Kinder-]DIPS-OA) with patients played by previously trained actors through video chat. All actors were blinded to the assigned teaching condition. Patient roles were based on 1 out of 3 case vignettes distributed evenly across courses, each for a different disorder (generalized anxiety disorder, obsessive-compulsive disorder, or major depression; “Section A” in Multimedia Appendix 2). Each case vignette included instructions to the actors to simulate difficult interview situations (eg, “Miss the point with your answer to this question.”; “Section A” in Multimedia Appendix 2). The interviews were videotaped and then rated by 4 blinded and independent evaluators using a coding scheme (“Section B” in Multimedia Appendix 2), which assessed 2 facets of interview performance—formal interviewing skills (10 items; eg, “The interviewer asks relevant additional questions beyond the interview guide to assess the presence of the diagnostic criteria.”) and interpersonal interviewing skills (9 items; eg, “The interviewer uses non-verbal and paraverbal interviewing techniques.”). Both dimensions were assessed on scales ranging from 0 to 100. To succeed in adequately conducting the structured interview, participants had to score at least 50% correct on both scales. The cutoff of 50% is commonly used in the German education system.

All the outcome assessors had a master’s degree in psychology, were certified and experienced conducting the (Kinder-)DIPS-OA, and received at least 2 years of postgraduate cognitive behavioral therapy training. Interrater reliability for each item was calculated based on 40 jointly coded interviews, with Fleiss κ ranging between fair (0.34) and almost perfect (0.96) agreement between outcome assessors [45].

#### Secondary Outcomes

##### Diagnostic Knowledge

Two parallel 15-item versions of a test of basic clinical diagnostic knowledge were created, which participants answered at t1 and t3 (refer to “Section C” in Multimedia Appendix 2 for example items). The format of the items varied (single choice, multiple select, and multiple-true-false) and the items were previously piloted with laypersons (30 undergraduate students in their first semester) and experts (44 therapists in postgraduate training). Items were selected based on item-scale correlation and discrimination between these 2 groups. In addition, at t1, the self-reported diagnostic knowledge was assessed on an 11-point Likert-type scale (“How knowledgeable are you in the area of ‘clinical diagnostics’?”; 0= “I don’t know anything about it”, 10= “I am very knowledgeable in this area”).

##### Participants’ Reactions

Participants’ reactions to the courses and the estimated patient acceptance of structured interviews were evaluated at t3 by means of an online questionnaire, which consisted of 32 selected items (Table 2) from several instruments [6,46-50]. There were 8 additional items only administered in the blended learning condition. Unless otherwise described, a 7-point Likert-type scale was used for the items, ranging from 1=“strongly disagree” to 7=“strongly agree”, with higher scores indicating a better outcome.

**Table 2:** Overview of the items assessing participants’ reactions.

Questionnaire and subscale			Items, n		Example		Cronbach α
**MFE-Sr^a^ [46]**							
	Intent to recommend	1		“I would recommend this course to other students.”		—^b^	
	Experience of overload	3		“The content of this course was too difficult for me.”^c^		0.77	
	Subjective learning success	1		“I learned a lot in this course.”		—	
**Web-CLIC^d^ [46]**							
	Clarity	3		“The contents of the course are clearly presented”		0.83	
	Likeability	3		“The course arouses my interest”		0.93	
	Informativeness	3		“The information is of high quality”		0.91	
	Credibility	3		“I can trust the information in the course”		0.95	
**Short scale for academic course evaluation [47]**							
	Course structure	3		“The course was clearly structured.”		0.73	
**UMUX-Lite^e^ [48]**							
	Usability	2		“This system is easy to use”^f^		0.82-0.83	
**VisAWI-S^g^ [49]**							
	Visual Aesthetics	4		“The layout is professional”^f^		0.76	
**Items designed by the study authors**							
	Visual Aesthetics	1		“DiSkO is designed to be visually appealing”^f^		—	
	Credibility	1		“I completely trusted the content in DiSkO”^f^		—	
	Overall impression	1		“Overall: I give the course an overall grade of …”^c,h^		—	
**Acceptance of structured interviews questionnaire [6]**							
	Global satisfaction rating	1		“Please indicate on the accompanying scale how satisfied you think patients are or would be with structured diagnostic interviews in general.”^i^		—	
	Mental effort and emotional reaction to structured interviews	10		“After a structured interview, patients feel more confused than before.”^j^		—	

^a^MFE-Sr: Münster Questionnaire for the Evaluation of Seminars – revised.

^b^Not applicable

^c^Lower scores indicate a better outcome.

^d^Web-CLIC: Website-Clarity, Likeability, Informativeness, and Credibility.

^e^UMUX-Lite: Usability Metric for User Experience – Lite.

^f^These items were only administered in the blended learning condition.

^g^VisAWI-S: Visual Aesthetics of Websites Inventory – Short.

^h^This item used the German grading system ranging from 1 (excellent) to 6 (insufficient).

^i^Visual analog scale ranging from 0 (not at all satisfied) to 100 (completely satisfied).

^j^4-point Likert scale ranging from 0 (disagree) to 3 (completely agree).

#### Statistical Analyses

All outcomes were evaluated at the individual participant level using noninferiority analyses. To assess noninferiority between the teaching conditions, 95% CIs were calculated. Although our hypothesis regarding the noninferiority of the blended learning course is inherently 1-sided, the use of a 2-sided 95% CI is standard practice [51] and recommended by the guidelines of the European Medicines Agency and the US Food and Drug Administration [52,53]. This approach allows for the interpretation of noninferiority if the entire 95% CI lies above the prespecified noninferiority margin.

A logistic regression was conducted, predicting the primary outcome (passing the performance test) based on the predictor’s teaching condition (blended learning vs synchronous), study site (center 1 vs center 2 vs center 3), course focus (adulthood vs childhood and adolescence), study year, self-reported diagnostic knowledge, and the score in the knowledge test at t1. To account for the effects of cluster randomization, the course variable was included as a random effect (random-intercept). Odds ratios (ORs) were calculated by unconditional maximum likelihood estimation and 95% CI using normal approximation. Based on experience with the traditional synchronous course format in diagnostic teaching, a passing rate of 85% was assumed for the synchronous course. As the passing rate after blended learning should be at least as good as that in traditional face-to-face instruction due to the positive effects of blended learning on learning outcomes [24], a 90% passing rate was assumed in the blended learning course. To test for noninferiority, the assumed passing rates and noninferiority margin of 5% were transferred to ORs [54,55]:


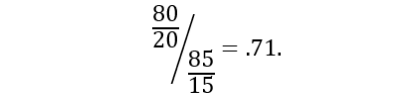


Accordingly, a power analysis for noninferiority trials with dichotomous data revealed for expected success rates of 85% and 90%, respectively; noninferiority margin of 5%; α=.05; and power of 80% a required sample size of 135 per treatment group [56].

All secondary outcome measures were tested using multiple linear regression models with the following predictors: teaching condition, study site, course focus, study year, self-reported diagnostic knowledge, and the score in the knowledge test at t1. Noninferiority of the blended learning course was assumed, when the lower bound of the CI of the predictor teaching condition was larger than β=–.10, corresponding to a small negative effect.

To test for systematic differences between teaching conditions at baseline, *t* tests and Fisher exact tests were conducted. Furthermore, we tested for differences in assigned teaching condition and practical diagnostic test performance between completers (participation in t1, t2, and t3) and noncompleters using chi-square tests.

All available data were analyzed for each statistical test performed. All analyses were run in R (R Core Team) [57]. The anonymized dataset [58] and R code [59] are available online.

### Ethical Considerations

The authors declare that all procedures contributing to this work comply with the ethical standards of the relevant national and institutional committees on human experimentation and with the Helsinki Declaration of 1975, as revised in 2008. This study protocol was reviewed and approved by the local ethics committee of the faculty of psychology at the Ruhr University Bochum (2021/686). Written informed consent was obtained from participants to participate in the study.

## Results

### Participant Characteristics at Baseline

**Figure 1:**
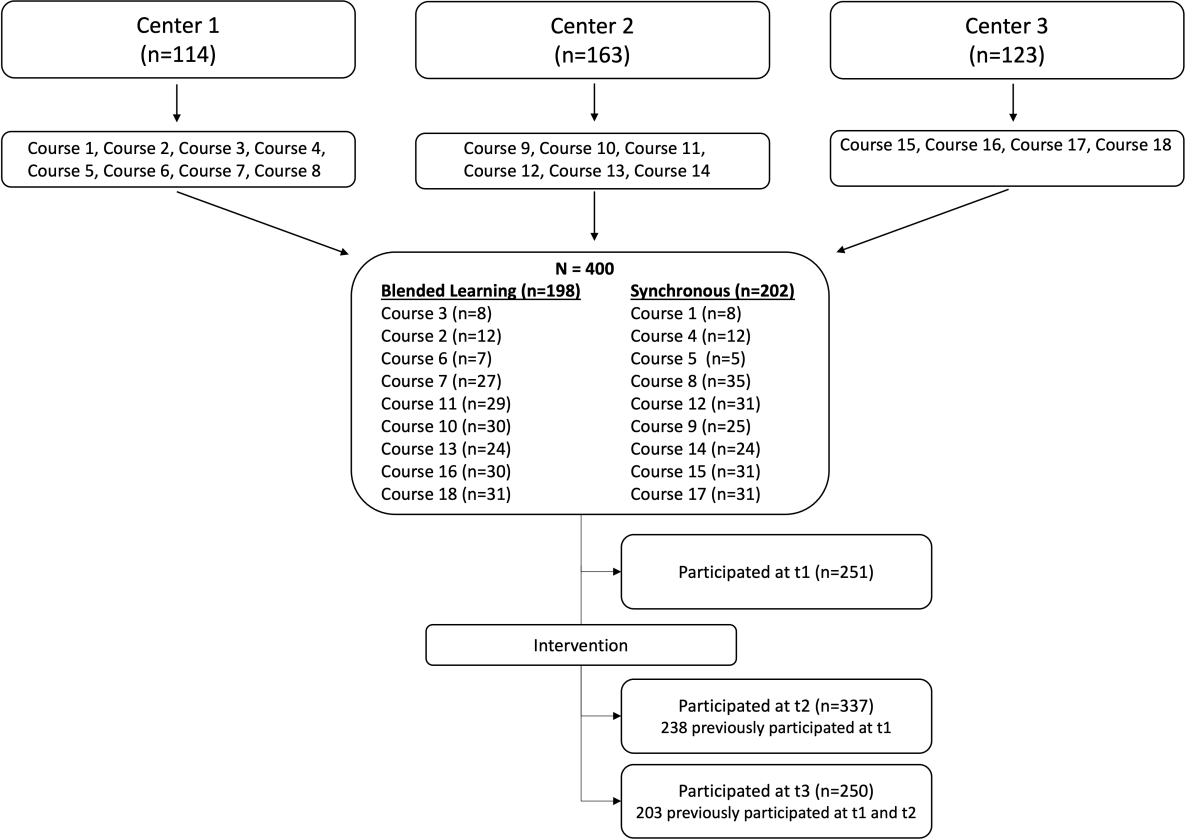
CONSORT (Consolidated Standards of Reporting Trials) flowchart of students enrolled in the courses and participating in the study.

Figure 1 shows the distribution of participants among the courses and the sample sizes at the measurement time points. A total of 350 participants took part in at least 1 of the 3 measurement time points, 203 of whom participated in all of them. Demographic data were missing from 17 participants because they did not complete the online survey that was part of the behavioral test. Participants (n=333) had a mean age of 23.6 (SD 4.52) years and the majority identified as women (279/333, 83.8%). The average study year was 2.82 (SD 0.93).

The *t* test and Fisher exact test revealed no significant differences between teaching conditions at baseline (all *P*>.05; Table 3). Chi-square tests showed no significant differences between completers and noncompleters regarding teaching condition (*χ*²_1_=0.79, *P*=.37) and behavioral performance (*χ*²_1_=0.82, *P*=.37).

**Table 3:** Participants’ baseline demographic characteristics and diagnostic knowledge.

Characteristic		Participants, n	Blended learning	Synchronous	*t* test (*df*)	*P* value
**Age (years), mean (SD)**		333	24.1 (4.20)	23.2 (4.76)	1.81 (331)	.07
**Study year, mean (SD)**		333	2.88 (0.93)	2.76 (0.94)	1.09 (331)	.28
**Diagnostic knowledge, mean (SD)**						
	Test score (t1)	251	9.44 (1.71)	9.36 (1.79)	0.37 (249)	.71
	Self-rating	333	3.46 (2.07)	3.51 (2.18)	–0.22 (331)	.82
**Gender, n (%)**		350				.05
	Missing		11 (6.47)	7 (3.89)	—^a^	
	Female		127 (74.71)	152 (84.44)	—	
	Male		31 (18.23)	18(10.0)	—	
	Diverse		1 (0.59)	3 (1.67)	—	

^a^Not applicable.

### Primary Outcome—Practical Diagnostic Skills

Overall, participants showed high levels of interpersonal (blended learning: mean 74.8, SD 16.2; synchronous: mean 70.7, SD 18.1) and formal skills (blended learning: mean 86.1, SD 14.4; synchronous: mean 82.8, SD 16.6). The passing rate was 89% in the blended learning condition and 74.6% in the synchronous condition, corresponding to an OR of 2.77 (95% CI 1.52-5.03).

We furthermore tested whether this finding still held up when several covariates were considered. For this, we fitted a logistic mixed model (adjusted model; n=320) including the course variable as random effect to take cluster randomization into account (Table 4; for all model coefficients, refer to “Section D” in Multimedia Appendix 2). As the model resulted in convergence errors with all predictors, the knowledge test score at t1 and the study center had to be excluded. More complex models accounting for the nesting of courses within universities were attempted to be fitted but resulted in convergence errors. Intraclass correlation is not reported as the results of the generalized linear mixed model did not contain the residual variance required to calculate the intraclass correlation.

**Table 4:** Odds ratio (OR) and 95% CI for the unadjusted and adjusted model.

Predictors		Unadjusted model, OR (95% CI)	Adjusted model, OR (95% CI)
**Teaching condition**		2.77 (1.55-5.13)	3.20 (1.56-6.71)
**Center 2**		—^a^	—
**Center 3**		—	—
**Course focus**		—	0.42 (0.17-0.96)
**Study year**		—	1.36 (0.82-2.42)
**Self-reported knowledge**		—	1.06 (0.89-1.25)
**Knowledge test (t1)**		—	—
**Random effects**			
	σ^2^	—	3.29
	N_course_	—	18
	Observations	337	320
	Tjur D	0.035	0.214

^a^Not applicable.

### Secondary Outcomes

#### Overview

To describe the magnitude of the differences in secondary outcomes between the groups, multiple linear regression models were calculated (Table 5). For the complete covariate-adjusted models, refer to “Section E” in Multimedia Appendix 2.

**Table 5:** Means, SDs, and β-coefficients with CIs for all secondary outcomes.

Secondary outcome		Range	Blended learning (n=117), mean (SD)	Synchronous (n=132), mean (SD)	Teaching condition, β (95% CI)
**Diagnostic knowledge**					
	Knowledge test (t3)	0-15	12.0 (1.65)	11.4 (1.68)	.13 (0.01 to 0.26)
**Participants’ reactions**					
	Intent to recommend	0-7	6.21 (1.06)	6.09 (1.13)	.09 (–0.05 to 0.22)
	Experience of overload^a^	0-7	2.70 (1.04)	2.40 (1.04)	.20 (0.07 to 0.34)
	Subjective learning success	0-7	5.89 (1.02)	5.80 (1.06)	.04 (–0.096 to 0.18)
	Clarity	0-7	6.18 (0.74)	5.48 (0.91)	.40 (0.27 to 0.53)
	Likeability	0-7	6.09 (0.96)	5.86 (1.21)	.09 (–0.04 to 0.23)
	Informativeness	0-7	6.38 (0.68)	6.19 (0.72)	.19 (0.06 to 0.33)
	Credibility	0-7	6.46 (0.64)	6.45 (0.62)	.08 (–0.05 to 0.22)
	Course structure	0-7	6.30 (0.72)	5.98 (0.92)	.18 (0.04 to 0.32)
	Overall impression	1-6	1.56 (0.81)	1.72 (0.75)	–.12 (–0.26 to 0.01)
	Visual Aesthetics	0-7	5.87 (0.91)	—^b^	—
	Usability	0-100	86.3 (14.1)	—	—
**Acceptance**					
	Global rating	0-100	77.7 (14.2)	76.8 (16.2)	.04 (–0.095 to 0.18)
	“More confused”^a^	0-3	0.26 (0.48)	0.42 (0.58)	–.11 (–0.25 to 0.03)
	“questioned out”^a^	0-3	1.18 (0.74)	1.20 (0.85)	.05 (–0.08 to 0.19)
	“too many questions”^a^	0-3	1.11 (0.81)	1.06 (0.76)	.03 (–0.11 to 0.16)
	“exhausting”^a^	0-3	1.15 (0.71)	1.07 (0.77)	.06 (–0.08 to 0.20)
	“taken seriously”	0-3	2.33 (0.88)	2.33 (0.84)	–.01 (–0.15 to 0.13)
	“positive relationship”	0-3	1.95 (0.80)	2.04 (0.75)	–.10 (–0.24 to 0.04)
	“not report everything”^a^	0-3	1.33 (0.81)	1.32 (0.86)	.04 (–0.10 to 0.18)
	“better understanding”	0-3	1.56 (0.76)	1.50 (0.80)	.07 (–0.07 to 0.21)
	“enough detail”	0-3	2.30 (0.75)	2.36 (0.72)	–.05 (–0.19 to 0.09)
	“helpful”	0-3	2.12 (0.74)	2.14 (0.72)	–.03 (–0.17 to 0.12)

^a^Lower scores indicate better outcome. For negatively-keyed items, noninferiority of the blended learning course can be assumed if the upper bound of the CI is greater than 0.10.

^b^Not applicable.

#### Diagnostic Knowledge

Participation in the blended learning course increased the knowledge score at t3 (β=.13, 95% CI 0.01-0.26). Thus, noninferiority and superiority of the blended learning course regarding diagnostic knowledge at t3 can be assumed.

#### Participants’ Reactions

Noninferiority of the blended learning course regarding participants’ reactions to the courses could be observed in most measures collected. Only with regard to the experience of overload the blended learning course was inferior to the synchronous course, with lower scores indicating a more favorable outcome (β=.20, 95% CI 0.07-0.34). Furthermore, the superiority of the blended learning over the synchronous course could be found in the following subscales: clarity (β=.40, 95% CI 0.27-0.53), course structure (β=.18, 95% CI 0.04-0.32), and informativeness (β=.19, 95% CI 0.06-0.32).

Regarding the estimated patient acceptance of structured interviews, noninferiority of the blended learning course was observed for the global acceptance rating (β=.04, 95% CI –0.095 to 0.18) and the items “After a structured interview, patients feel more confused than before” (β=–0.11, 95% CI –0.25 to 0.03) and “Patients have the feeling that they understand themselves and their problems better, after a structured interview” (β=.07, 95% CI –0.07 to 0.21). For the other items, student’s estimation did not differ between the blended learning and the synchronous courses.

## Discussion

### Principal Findings

The aim of the present study was to establish whether a blended learning course with a reduced personal contact time results in comparable clinical diagnostic skills as a traditional synchronous online course.

The results of this study are in line with and extend the existing literature on blended learning [24,35,36]. First, noninferiority and superiority of the blended learning course over the synchronous course could be found for the primary outcome measure—the performance in a simulated structured diagnostic interview. Second, noninferiority and superiority were also observed for the diagnostic knowledge test score at t3 and several reaction measures, such as clarity, informativeness, and structure of the course. Furthermore, noninferiority could be observed regarding the intention to recommend the course to other students, subjective learning success, likeability, credibility, overall impression of the course, and 3 items of the estimated patient acceptance of structured clinical interviews. Third, inferiority of the blended learning compared with the synchronous course was found for the participants’ experience of overload.

Despite the described differences, participants in both courses showed high levels of interpersonal and formal skills, good diagnostic knowledge, positive reactions to the courses, and high-estimated patient acceptance. While therapists were found to underestimate patient acceptance of structured interviews [6], estimated patient acceptance ratings in this study correspond more closely to patients’ actual acceptance ratings [60], indicating that participants of the present study estimated patient acceptance more accurately than did therapists in the aforementioned study.

### Limitations and Strengths

The study has some limitations that should be mentioned. First, the blended learning course was presented as a block, meaning that participants had 3 weeks of time to work through the asynchronous online content followed by 3 weekly synchronous online sessions. In contrast, the synchronous online course consisted of 11 weekly sessions. The fact that participants only had 3 weeks for the online content, which was equivalent to 8 sessions of 90 minutes each, might be considered a disadvantage for the blended learning course. This might explain why participants in the blended learning course reported higher levels of overload than those in the synchronous online course. To reduce the experience of overload and possibly even further enhance students’ performance, the blended learning course should be provided on a continuous basis in the future, ensuring continuous student activity [61]. Second, adherence in the control condition was not assessed. Although teachers were informed about the mandatory content in a training session and received course material before the start of the course, it remains unclear whether all mandatory content was in fact taught in the control condition. In contrast, the asynchronous online component of the blended learning course was meticulously developed. It was ensured that the blended learning course contained all the necessary content for the diagnostic skills and knowledge tests. In addition, formative evaluations were conducted to assess the content, usability, and design of the course. Based on the feedback received, the course was subsequently revised. Therefore, the blended course was designed and implemented with more effort than the control condition, which followed a “teaching as usual” rationale. Third, we did not evaluate how the participants used the courses and materials for preparation, repetition, and reflection of the lectures. The convenient repetition offered by the asynchronous materials in the blended-learning course may have been particularly beneficial for the practical skills and knowledge tests. Fourth, all synchronous sessions in both the experimental and control conditions had to be conducted online due to governmental restrictions associated with the COVID-19 pandemic. As a result, the experimental condition had to be adapted to a hybrid combination of synchronous and asynchronous online teaching, although it was initially intended to be a combination of synchronous face-to-face teaching and asynchronous online teaching. Thus, it could be argued that our experimental condition does not strictly qualify as blended learning since it did not include face-to-face teaching. To evaluate the potential implications of this adaption, it is important to consider several differences between synchronous online and offline teaching. While both provide the benefit of immediate educational support and feedback [62], online teaching has logistical, instructional, and financial benefits over offline teaching [63]. However, online learning’s logistical flexibility also has the potential downside of causing social isolation for the learner [64]. In addition, the integration of technology in educational settings inevitably increases the likelihood of technical issues, which can decrease satisfaction and participation [64,65]. Recent meta-analyses suggest that there are no significant differences in learning success between online and offline teaching [19,20]. Furthermore, online teaching received significantly higher satisfaction ratings compared with offline teaching [19]. It is important to note that our blended learning course primarily consisted of asynchronous online sessions. It can be assumed that the impact of 3 synchronous sessions, regardless of whether they are taught online or offline, is relatively small. However, it remains unclear how the blended learning course would compare with a traditional face-to-face course.

Besides these limitations, the study also has some notable strengths. First, as a multicenter cluster randomized controlled trial, it makes an important contribution to the scarce evidence on the efficacy of blended learning in general [33-35] and, more specifically, for teaching evidence-based diagnostics. Second, the design of the evaluation study was developed very carefully. For instance, the outcome measures were assessed with reliability and validity in mind, the case vignettes for students’ performance tests included very precise instructions, and actors were trained beforehand to ensure a high standardization. In addition, the items of the knowledge test were piloted on laypersons and therapists. Third, as undergraduate psychology students from 3 German universities attending a mandatory seminar of the diagnostics of mental disorders were invited to participate, a large sample of 337 participants could be included in the analysis of the primary outcome and 203 participants took part at all 3 measurement time points. Fourth, as the study was conducted in an ongoing university setting, a high external validity and generalizability of the study results can be assumed.

### Clinical Implications and Future Research

As the results indicate that the blended learning course can be used to teach evidence-based diagnostics, we aim to disseminate the blended learning course open access throughout Germany—at universities (undergraduate and graduate courses), at institutions of tertiary education, and among practicing psychotherapists. In order to facilitate the adoption of the blended learning course [66], a technical infrastructure was chosen which is available free of charge and provides ongoing technical support. In addition, an interesting question for future research is whether structured interviews are in fact used more frequently after attending the blended learning course. Increasing the use of structured interviews in clinical practice is an important goal as therapists appeared to use structured interviews only with 14.8% (55/370) of their patients [6]. Until today, research on therapist training is limited, especially when it comes to web-based training [67]. Therefore, to extend the promising findings of this study, future research should also focus on the development and evaluation of further blended learning courses to improve evidence-based practice in clinical psychology in general.

### Conclusion

In conclusion, this study suggests that a blended learning course, compared with a synchronous online course in a cluster randomized controlled trial, can be used to efficiently teach evidence-based diagnostics. The results indicate that the blended learning approach was more effective than synchronous online teaching in acquiring practical diagnostic skills and diagnostic knowledge, and that it was well received by the students. The blended learning course can therefore help to improve the skills and knowledge of (future) clinicians in a time- and cost-efficient way and thus make an important contribution to improving the diagnostics of mental disorders and the mental health care situation in the long term.

## Appendix

Multimedia Appendix 1 CONSORT-eHEALTH checklist (V 1.6.x).

Multimedia Appendix 2 Case vignette and instructions for the actors, coding scheme, knowledge test, logistic regression coefficients for the primary outcome, and multiple linear regression coefficients for secondary outcomes.

## Abbreviations

DIPS-OA1.2: Diagnostic Interview for Mental Disorders – Open Access 1.2
(Kinder-)DIPS-OA: Diagnostic Interview for Mental Disorders (in Children and Adolescents) – Open Access
OR: odds ratio
