# Supplementary material for: A Blended Learning Course on the Diagnostics of Mental Disorders: Multicenter Cluster Randomized Noninferiority Trial
Source: J Med Internet Res. 2024 Nov 27;26:e54176. doi: 10.2196/54176 (PMC11635323; doi:10.2196/54176)
Supplement: Multimedia Appendix 2 [file jmir_v26i1e54176_app2.docx]

Contents

[Section A: Case vignette and instructions for the actors 2](#_Toc163131291)

Section [B: Coding scheme 4](#_Toc163131292)

[Section C: Knowledge Test 6](#_Toc163131293)

Section [D: Logistic regression coefficients for the primary outcome 7](#_Toc163131294)

[Section E: Multiple linear regression coefficients for secondary outcomes 9](#_Toc163131295)

## Section A: Case vignette and instructions for the actors

**Case vignette: Generalized Anxiety Disorder**

Your name is Nico Schreiber, you are 27 years old and you live together with your girlfriend and a cat in a rented apartment. You finished your teacher training course last year and since then you do your traineeship at a secondary modern school.

For the last 1.5 years you went to your primary care physician repeatedly because of continuous difficulties sleeping (mainly problems to fall asleep), restlessness, muscle tension and a persistent headache. You suffer from these symptoms most of the days and you experience these symptoms as very serious.

Medical examinations (among other things full blood count and MRI) did not reveal anything. Your physician’s recommendations (e.g., regular exercise like jogging or walking in the forest), stress reduction or heat plasters to reduce tension of shoulders and neck) did not result in a real improvement.

At the last appointment with your physician, you told her that you always worried a lot about everything imaginable. However, for some time you feel that your worries and anxiety even increased and that the worries determine your everyday life more and more. She recommended to contact a psychotherapist.

As the worries started slowly you cannot tell when they became that pronounced and burdensome. There was no specific trigger. You guess that it was at the end of your study, about one year ago. Since then, your worries intrude repeatedly and you can hardly concentrate on anything else (e.g., reading or watching a series).

The topics that you worry about are very diverse. You run through scenarios over and over again, without finding a solution and your thoughts jump from one worry to another. For example, you worry that one of your family members (parents, sister, girlfriend) could have an accident or that somebody could become seriously ill, you question your professional competence and worry about your professional and financial future. Additionally, you worry about everyday things. Therefore, you try to plan your everyday life very thoroughly to, e.g., avoid being late at work or at an appointment. All in all, you feel worried and tense for at least 70% of the day.

You feel strongly impaired by the worries, because, for example, you have difficulties concentrating at work and find school life very exhausting, although you actually like working with children. In addition, you cannot enjoy your free time. For instance, you cancel appointments with friends or activities with your girlfriend, because you slept badly and/or have neck pain or a headache. Furthermore, you feel strongly burdened by your worries, because you are nearly never able to calm down and be free of worries.

You did not take any medication, drugs or alcohol shortly before the symptoms began and you did not have any physical illnesses that might have caused the symptoms.

You do not have any attack-like anxiety symptoms like heart racing or shortness of breath. The worries cannot be reduced by repeated actions or compulsions (e.g., frequent hand washing for fear of getting ill).

**Instructions for the actors: Generalized Anxiety Disorder**

*In order to include some difficult situations as standardized as possible, we specify for a few questions which answer should be given. All other questions can be answered according to the case vignette without following additional instructions.*

**Interview question *1.1:** *In the last 6 months, on most days, have you been very worried and anxious about various things or everyday matters?*

RESPONSE: "I worry about all kinds of things, such as about my family members, about my job future ... but also about being late for work or an appointment."

(Anticipate answers to later questions).

**Interview question *1.3:** *Is it difficult for you to control or stop worrying, or do your worries crowd you when you are trying to focus on something else (e.g., reading to distract yourself)?*

RESPONSE: "What do you mean by controlling my worries?"

After the interviewer explanation:

RESPONSE: "Yes." (No further explanation, only when asked).

**Interview question *3.5**: Are you worried about your own health?

RESPONSE: "Yes, I am constantly worried that my partner will get seriously ill or have an accident." (Answer question "wrong" / talk out of turn).

## Section B: Coding scheme

| **Coding:**  1=fulfilled  0=not fulfilled  99=irrelevant | **Total**  **score** | | | | | |
| --- | --- | --- | --- | --- | --- | --- |
|  | **code** |  |  |  |  |  |
|  | **time** |  |  |  |  |  |
| 1. **Formal interviewing skills** | | | | | | |
| - 1. The interviewer rephrases the questions in the interview guide appropriately in case of comprehension problems. |  | | | | | |
|  | **code** |  |  |  |  |  |
|  | **time** |  |  |  |  |  |
|  |  | | | | | |
| - 1. The interviewer notices when the patient misses the point of the question and rephrases it. |  | | | | | |
|  | **code** |  |  |  |  |  |
|  | **time** |  |  |  |  |  |
|  |  | | | | | |
| - 1. The interviewer refers to previous statements made by the patient (e.g., adjusts or skips questions if the respective information has already been asked). |  | | | | | |
|  | **code** |  |  |  |  |  |
|  | **time** |  |  |  |  |  |
|  |  | | | | | |
| - 1. The interviewer asks relevant additional questions beyond the interview guide to assess the presence of the diagnostic criteria. |  | | | | | |
|  | **code** |  |  |  |  |  |
|  | **time** |  |  |  |  |  |
|  |  | | | | | |
| - 1. The interviewer explores in detail the patient's impairment and burden of symptoms. |  | | | | | |
|  | **code** |  |  |  |  |  |
|  | **time** |  |  |  |  |  |
|  |  | | | | | |
| - 1. The interviewer asks all relevant criterion questions from the interview guide. |  | | | | | |
|  | **code** |  |  |  |  |  |
|  | **time** |  |  |  |  |  |
|  |  | | | | | |
| - 1. The interviewer explores lifetime symptoms only when symptoms are currently not present. |  | | | | | |
|  | **code** |  |  |  |  |  |
|  | **time** |  |  |  |  |  |
|  |  | | | | | |
| - 1. The interviewer rephrases questions appropriately so that the relevant information is collected. |  | | | | | |
|  | **code** |  |  |  |  |  |
|  | **time** |  |  |  |  |  |
|  |  | | | | | |
| - 1. The interviewer provides only verbal and no numerical anchors for scaled questions |  | | | | | |
|  | **code** |  |  |  |  |  |
|  | **time** |  |  |  |  |  |
|  |  | | | | | |
| - 1. The interviewer reads out only those questions in the interview guide that are also addressed to the patient, and not the instructions to the interviewer. |  | | | | | |
|  | **code** |  |  |  |  |  |
|  | **time** |  |  |  |  |  |
|  |  | | | | | |
| 1. **Interpersonal interviewing skills** | | | | | | |
| - 1. The interviewer paraphrases what the patient says when it seems appropriate. |  | | | | | |
|  | **code** |  |  |  |  |  |
|  | **time** |  |  |  |  |  |
|  |  | | | | | |
| - 1. The interviewer politely interrupts the patient if he/she digresses from answering the question. |  | | | | | |
|  | **code** |  |  |  |  |  |
|  | **time** |  |  |  |  |  |
|  |  | | | | | |
| - 1. The interviewer avoids asking leading questions. |  | | | | | |
|  | **code** |  |  |  |  |  |
|  | **time** |  |  |  |  |  |
|  |  | | | | | |
| - 1. The interviewer does not allow a long pause in the interview (at least 30 seconds) to occur without informing the patient. |  | | | | | |
|  | **code** |  |  |  |  |  |
|  | **time** |  |  |  |  |  |
|  |  | | | | | |
| - 1. The interviewer uses non-verbal and paraverbal interviewing techniques (e.g., nodding, maintaining eye contact) |  | | | | | |
|  | **code** |  |  |  |  |  |
|  | **time** |  |  |  |  |  |
|  |  | | | | | |
| - 1. The interviewer structures the conversation (e.g. "we'll talk about this again in more detail at another point"). |  | | | | | |
|  | **code** |  |  |  |  |  |
|  | **time** |  |  |  |  |  |
|  |  | | | | | |
| - 1. The interviewer expresses understanding at the right time or validates emotions arising in the patient. |  | | | | | |
|  | **code** |  |  |  |  |  |
|  | **time** |  |  |  |  |  |
|  |  | | | | | |
| - 1. The interviewer encourages or thanks the patient when it seems appropriate (e.g., "You're doing really well", "Thank you for your detailed answer") |  | | | | | |
|  | **code** |  |  |  |  |  |
|  | **time** |  |  |  |  |  |
|  |  | | | | | |
| - 1. The interviewer shows a naive and non-judgmental attitude. |  | | | | | |
|  | **code** |  |  |  |  |  |
|  | **time** |  |  |  |  |  |
|  |  | | | | | |

## Section C: Knowledge Test

**Example Items**

1. Different anxiety disorders are best distinguished from each other by _______________. *Please choose the correct term*:
2. Avoidance of typical situations
3. **underlying fears**
4. learning history
5. distinct conditioning processes
6. distinctive learning
7. Peter has been neglected by his parents for years. They left him alone in the apartment or did not provide enough food. Now he is 8 years old and suffers from nightmares, low self-esteem and problems trusting other people as well as from eating attacks where he regularly overeats. According to the DSM-5, why doesn't he have PTSD? *Several answers may be correct.*
8. **The A criterion (confrontation with actual or threatened death, sexual violence) is not met.**
9. He has an eating disorder.
10. He has a depressive disorder.
11. **The number of symptoms is not sufficient. For example, symptoms of the C criterion (avoidance of stimuli related to the trauma) or the E criterion (changes in arousal level such as startle or the like) are missing.**
12. The memory of young children is not mature enough to form a trauma memory as required for PTSD.
13. A patient does not want to share the content of his thoughts. He is visibly ashamed of them. Is this more likely to represent worry (in the context of a GAD) or obsessive thoughts?

**Obsessive thoughts**

## Section D: Logistic regression coefficients for the primary outcome

Additionally, to the two reported models in the main manuscript (unadjusted model and adjusted model 3), we fitted two more binomial logistic regression models (adjusted models 1 and 2) to predict the likelihood of passing the behavioral test (see Table S1):

The first adjusted model (*n*=238) included teaching condition (reference category: synchronous), study site (reference category: center 1), course focus (reference category: childhood and adolescence), study year, self-reported diagnostic knowledge, and the knowledge test score (t1) as predictors.

The second adjusted model (*n*=320) was calculated as a trade-off between number of missing values and controlling variables, using all predictors of the first adjusted model except the knowledge test score at t1.

In all four models, the lower bound of the 95% CI for the odds of passing the behavioral test were larger than the pre-specified non-inferiority margin of .71, indicating non-inferiority of the blended learning course. As shown in Figure S1, the blended learning course was not only not inferior to the synchronous course in all four models, but superior.

Figure S1. OR with 95% CI for the predictor teaching condition of all models


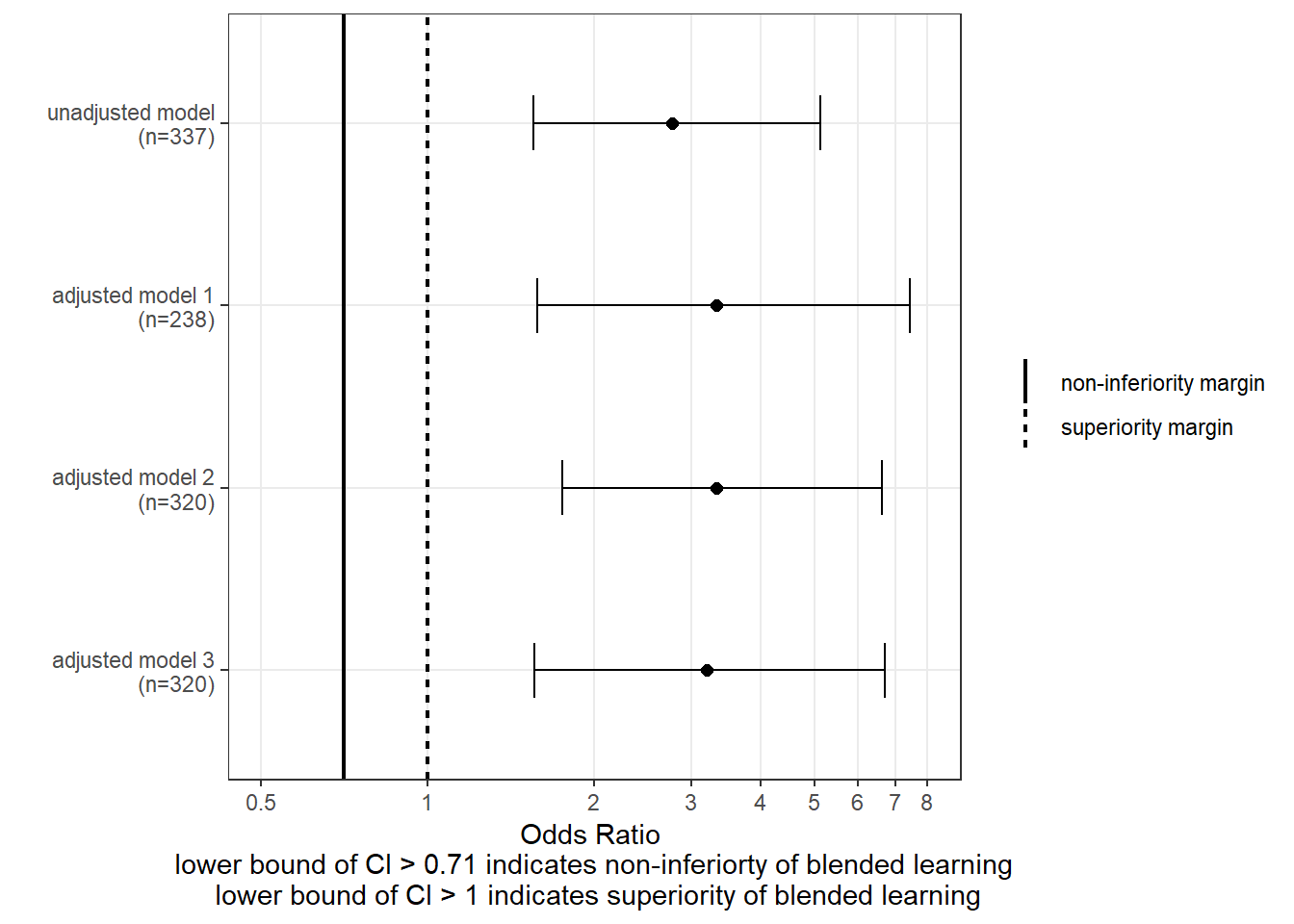


Table S1. Coefficients, OR and 95% CI for models 1-4

|  |  | *B* | *SE* | *z value* | *p* | *OR* | *95% CI for OR* | |
| --- | --- | --- | --- | --- | --- | --- | --- | --- |
|  |  |  |  |  |  |  | *lower* | *upper* |
| Model 1 | Constant | 1.08 | .18 | 6.16 | < .001 |  |  |  |
|  | Teaching condition | 1.02 | .31 | 3.34 | < .001 | 2.77 | 1.55 | 5.13 |
|  | Tjur’s D=.035 ; *n*=337 | | | | | | | |
| Model 2 | Constant | -1.49 | 1.33 | -1.12 | .263 |  |  |  |
|  | Teaching condition | 1.20 | .39 | 3.07 | .002 | 3.33 | 1.58 | 7.45 |
|  | Center 2 | 1.40 | .87 | 1.61 | .108 | 4.06 | .727 | 23.2 |
|  | Center 3 | -.14 | .41 | -.34 | .733 | .868 | .382 | 1.95 |
|  | Course focus | -.06 | .62 | -.10 | .924 | .943 | .258 | 3.05 |
|  | Study year | -.12 | .17 | -.71 | .480 | .886 | .634 | 1.25 |
|  | Self-reported knowledge | .10 | .11 | .86 | .391 | 1.10 | .888 | 1.38 |
|  | Knowledge test (t1) | .29 | .12 | 2.34 | .020 | 1.33 | 1.05 | 1.70 |
|  | Tjur’s D=.146; *n*=238 | | | | | | | |
| Model 3 | Constant | .19 | .84 | .23 | .821 |  |  |  |
|  | Teaching condition | 1.20 | .34 | 3.56 | < .001 | 3.33 | 1.75 | 6.63 |
|  | Center 2 | 1.53 | .74 | 2.07 | .038 | 4.62 | 1.05 | 19.6 |
|  | Center 3 | -.18 | .36 | -.50 | .617 | .836 | .411 | 1.69 |
|  | Course focus | .14 | .58 | .24 | .810 | 1.15 | .337 | 3.46 |
|  | Study year | .05 | .14 | .35 | .728 | 1.05 | .804 | 1.40 |
|  | Self-reported knowledge | .04 | .08 | .54 | .591 | 1.04 | .891 | 1.23 |
|  | Tjur’s D=.116; *n*=320 | | | | | | | |
| Model 4 | Constant | .60 | .86 | .70 | .483 |  |  |  |
|  | Teaching condition | 1.16 | .34 | 3.46 | < .001 | 3.20 | 1.56 | 6.71 |
|  | Course forcus | .88 | .44 | 2.02 | .043 | 0.42 | 0.17 | 0.96 |
|  | Study year | .31 | .28 | 1.10 | .271 | 1.36 | 0.82 | 2.42 |
|  | Self reported knowledge | .06 | .09 | .68 | .499 | 1.06 | 0.89 | 1.25 |
|  | Tjur’s D=.214; *n*=320 | | | | | | | |

## Section E: Multiple linear regression coefficients for secondary outcomes

**Diagnostic knowledge** (*df*=195, *n*=203)

|  |  | *SE* | *t value* | *p* | *β* | *95% CI for β* | |
| --- | --- | --- | --- | --- | --- | --- | --- |
|  |  |  |  |  |  | *lower* | *upper* |
| Knowledge | Constant | .00 | 8.92 | <.001 | .00 | .00 | .00 |
|  | Teaching condition | .06 | 2.09 | .037 | .13 | .01 | .26 |
|  | Center 2 | .14 | 1.67 | .096 | .23 | -.04 | .50 |
|  | Center 3 | .08 | .36 | .717 | .03 | -.13 | .18 |
|  | Course focus | .12 | 1.91 | .058 | .23 | -.01 | .46 |
|  | Study year | .10 | 1.01 | .315 | .10 | -.09 | .29 |
|  | Self-reported diagnostic knowledge | .08 | .69 | .488 | .05 | -.10 | .20 |
|  | Knowledge test (t1) | .07 | 4.37 | < .001 | .33 | .18 | .47 |

**Participants’ reactions** (*df*=195, *n*=203)

|  |  | *SE* | *t value* | *p* | *β* | *95% CI for β* | |
| --- | --- | --- | --- | --- | --- | --- | --- |
|  |  |  |  |  |  | *lower* | *upper* |
| Intent to recommend | Constant | .00 | 10.71 | <.001 | .00 | .00 | .00 |
|  | Teaching condition | .07 | 1.23 | .219 | .09 | -.05 | .22 |
|  | Center 2 | .15 | -2.50 | .013 | -.37 | -.66 | -.08 |
|  | Center 3 | .09 | -1.29 | .197 | -.11 | -.28 | .06 |
|  | Course focus | .13 | -.95 | .343 | -.12 | -.38 | .13 |
|  | Study year | .11 | -.44 | .658 | -.05 | -.25 | .16 |
|  | Self-reported diagnostic knowledge | .08 | -.78 | .435 | -.06 | -.23 | .10 |
|  | Knowledge test (t1) | .08 | 2.06 | .041 | .17 | .01 | .33 |
| Subjective learning success | Constant | .00 | 10.28 | <.001 | .00 | .00 | .00 |
|  | Teaching condition | .07 | .59 | .554 | .04 | -.096 | .18 |
|  | Center 2 | .15 | -1.84 | .068 | -.27 | -.57 | .02 |
|  | Center 3 | .09 | -2.81 | .005 | -.24 | -.41 | -.07 |
|  | Course focus | .13 | -.25 | .802 | -.03 | -.29 | .22 |
|  | Study year | .11 | -.30 | .765 | -.03 | -.24 | .18 |
|  | Self-reported diagnostic knowledge | .08 | -1.07 | .285 | -.09 | -.25 | .07 |
|  | Knowledge test (t1) | .08 | 2.03 | .044 | .16 | .004 | .33 |
| Experience of overload | Constant | .00 | 6.42 | <.001 | .00 | .00 | .00 |
|  | Teaching condition | .07 | 3.00 | .003 | .20 | .07 | .34 |
|  | Center 2 | .15 | 1.12 | .265 | .16 | -.12 | .45 |
|  | Center 3 | .08 | 2.21 | .028 | .18 | .02 | .35 |
|  | Course focus | .13 | .15 | .881 | .02 | -.23 | .27 |
|  | Study year | .10 | -2.26 | .025 | -.23 | -.44 | -.03 |
|  | Self-reported diagnostic knowledge | .08 | 1.63 | .106 | .13 | -.03 | .29 |
|  | Knowledge test (t1) | .08 | -1.76 | .080 | -.14 | -.30 | .02 |

|  |  | *SE* | *t value* | *p* | *β* | *95% CI for β* | |
| --- | --- | --- | --- | --- | --- | --- | --- |
|  |  |  |  |  |  | *lower* | *upper* |
| Overall impression | Constant | .00 | 5.05 | <.001 | .00 | .00 | .00 |
|  | Teaching condition | .07 | -1.79 | .075 | -.12 | -.26 | .01 |
|  | Center 2 | .15 | 3.56 | <.001 | .52 | .23 | .81 |
|  | Center 3 | .09 | 2.22 | .028 | .19 | .02 | .35 |
|  | Course focus | .13 | 1.31 | .191 | .17 | -.08 | .42 |
|  | Study year | .10 | -.99 | .326 | -.10 | -.31 | .10 |
|  | Self-reported diagnostic knowledge | .08 | 1.37 | .172 | .11 | -.05 | .27 |
|  | Knowledge test (t1) | .08 | -2.20 | .029 | -.18 | -.33 | -.02 |
| Course structure | Constant | .00 | 14.05 | <.001 | .00 | .00 | .00 |
|  | Teaching condition | .07 | 2.51 | .013 | .18 | .04 | .32 |
|  | Center 2 | .15 | -.59 | .556 | -.09 | -.39 | .21 |
|  | Center 3 | .09 | -1.36 | .176 | -.12 | -.29 | .05 |
|  | Course focus | .13 | -.63 | .532 | -.08 | -.34 | .18 |
|  | Study year | .11 | -1.06 | .289 | -.11 | -.32 | .10 |
|  | Self-reported diagnostic knowledge | .08 | -.04 | .966 | -.004 | -.17 | .16 |
|  | Knowledge test (t1) | .08 | 1.15 | .251 | .09 | -.07 | .26 |
| Likeability | Constant | .00 | 10.46 | <.001 | .00 | .00 | .00 |
|  | Teaching condition | .07 | 1.36 | .175 | .09 | -.04 | .23 |
|  | Center 2 | .15 | -3.24 | .001 | -.47 | -.76 | -.19 |
|  | Center 3 | .08 | -.61 | .543 | -.05 | -.22 | .11 |
|  | Course focus | .13 | -1.34 | .181 | -.17 | -.42 | .08 |
|  | Study year | .10 | -.05 | .959 | -.01 | -.21 | .20 |
|  | Self-reported diagnostic knowledge | .08 | -1.01 | .316 | -.08 | -.24 | .08 |
|  | Knowledge test (t1) | .08 | 1.90 | .059 | .15 | -.01 | .31 |

|  |  | *SE* | *t value* | *p* | *β* | *95% CI for β* | |
| --- | --- | --- | --- | --- | --- | --- | --- |
|  |  |  |  |  |  | *lower* | *upper* |
| Credibility | Constant | .00 | 21.62 | <.001 | .00 | .00 | .00 |
|  | Teaching condition | .07 | 1.20 | .233 | .08 | -.05 | .22 |
|  | Center 2 | .15 | .04 | .964 | .01 | -.29 | .30 |
|  | Center 3 | .09 | -3.13 | .002 | -.27 | -.43 | -.10 |
|  | Course focus | .13 | .24 | .813 | .03 | -.23 | .29 |
|  | Study year | .11 | -1.21 | .228 | -.13 | -.34 | .08 |
|  | Self-reported diagnostic knowledge | .08 | -1.31 | .193 | -.11 | -.27 | .05 |
|  | Knowledge test (t1) | .08 | -.54 | .590 | -.04 | -.20 | .12 |
| Informativeness | Constant | .00 | 17.76 | <.001 | .00 | .00 | .00 |
|  | Teaching condition | .07 | 2.87 | .005 | .19 | .06 | .33 |
|  | Center 2 | .14 | -2.17 | .031 | -.31 | -.60 | -.03 |
|  | Center 3 | .08 | -2.13 | .034 | -.18 | -.34 | -.01 |
|  | Course focus | .13 | -.68 | .497 | -.09 | -.33 | .16 |
|  | Study year | .10 | -.40 | .686 | -.04 | -.24 | .16 |
|  | Self-reported diagnostic knowledge | .08 | -2.25 | .025 | -.18 | -.34 | -.02 |
|  | Knowledge test (t1) | .08 | 1.51 | .133 | .12 | -.04 | .27 |
| Clarity | Constant | .00 | 14.45 | <.001 | .00 | .00 | .00 |
|  | Teaching condition | .06 | 6.17 | <.001 | .40 | .27 | .53 |
|  | Center 2 | .14 | -1.49 | .139 | -.21 | -.48 | .07 |
|  | Center 3 | .08 | -1.36 | .175 | -.11 | -.26 | .05 |
|  | Course focus | .12 | -1.12 | .265 | -.13 | -.37 | .10 |
|  | Study year | .10 | -.89 | .375 | -.09 | -.28 | .11 |
|  | Self-reported diagnostic knowledge | .08 | .16 | .871 | .01 | -.14 | .16 |
|  | Knowledge test (t1) | .08 | -1.05 | .295 | -.08 | -.23 | .07 |

**Acceptance** (*df*=195, *n*=203)

|  |  | *SE* | *t value* | *p* | *β* | *95% CI for β* | |
| --- | --- | --- | --- | --- | --- | --- | --- |
|  |  |  |  |  |  | *lower* | *upper* |
| Global rating | Constant | .00 | 8.70 | <.001 | .00 | .00 | .00 |
|  | Teaching condition | .07 | .59 | .553 | .04 | -.10 | .18 |
|  | Center 2 | .15 | -1.20 | .230 | -.18 | -.47 | .11 |
|  | Center 3 | .08 | -3.96 | <.001 | -.33 | -.50 | -.17 |
|  | Course focus | .13 | .18 | .858 | .02 | -.23 | .28 |
|  | Study year | .10 | -.49 | .628 | -.05 | -.26 | .16 |
|  | Self-reported diagnostic knowledge | .08 | -.19 | .846 | -.02 | -.18 | .14 |
|  | Knowledge test (t1) | .08 | 1.96 | .051 | .16 | -.001 | .32 |
| “more confused” | Constant | .00 | 2.42 | .016 | .00 | .00 | .00 |
|  | Teaching condition | .07 | -1.59 | .114 | -.11 | -.25 | .03 |
|  | Center 2 | .15 | -.78 | .434 | -.12 | -.41 | .18 |
|  | Center 3 | .08 | 2.42 | .017 | .21 | .04 | .37 |
|  | Course focus | .13 | -1.02 | .308 | -.13 | -.39 | .12 |
|  | Study year | .11 | .95 | .343 | .10 | -.11 | .31 |
|  | Self-reported diagnostic knowledge | .08 | -.47 | .639 | -.04 | -.20 | .12 |
|  | Knowledge test (t1) | .08 | -1.69 | .093 | -.14 | -.30 | .02 |
| “questioned out” | Constant | .00 | 4.45 | <.001 | .00 | .00 | .00 |
|  | Teaching condition | .07 | .77 | .445 | .05 | -.08 | .19 |
|  | Center 2 | .15 | -.04 | .969 | -.01 | -.29 | .28 |
|  | Center 3 | .08 | 2.72 | .007 | .23 | .06 | .39 |
|  | Course focus | .13 | -.89 | .372 | -.11 | -.36 | .14 |
|  | Study year | .10 | -.42 | .676 | -.04 | -.25 | .16 |
|  | Self-reported diagnostic knowledge | .08 | -2.17 | .031 | -.17 | -.33 | -.02 |
|  | Knowledge test (t1) | .08 | -.99 | .326 | -.08 | -.24 | .08 |

|  |  | *SE* | *t value* | *p* | *β* | *95% CI for β* | |
| --- | --- | --- | --- | --- | --- | --- | --- |
|  |  |  |  |  |  | *lower* | *upper* |
| “too many questions” | Constant | .00 | 3.93 | <.001 | .00 | .00 | .00 |
|  | Teaching condition | .07 | .36 | .718 | .03 | -.11 | .16 |
|  | Center 2 | .15 | .08 | .934 | .01 | -.28 | .31 |
|  | Center 3 | .09 | 2.37 | .019 | .20 | .03 | .37 |
|  | Course focus | .13 | -1.34 | .183 | -.17 | -.43 | .08 |
|  | Study year | .11 | 1.29 | .197 | .14 | -.07 | .35 |
|  | Self-reported diagnostic knowledge | .08 | .09 | .927 | .01 | -.15 | .17 |
|  | Knowledge test (t1) | .08 | -2.38 | .018 | -.19 | -.35 | -.03 |
| “exhausting” | Constant | .00 | 3.07 | .002 | .00 | .00 | .00 |
|  | Teaching condition | .07 | .84 | .404 | .06 | -.08 | .20 |
|  | Center 2 | .15 | -.57 | .568 | -.09 | -.39 | .21 |
|  | Center 3 | .09 | 1.01 | .312 | .09 | -.08 | .26 |
|  | Course focus | .13 | -.23 | .820 | -.03 | -.29 | .23 |
|  | Study year | .11 | .66 | .511 | .07 | -.14 | .29 |
|  | Self-reported diagnostic knowledge | .08 | .35 | .724 | .03 | -.14 | .20 |
|  | Knowledge test (t1) | .08 | -1.04 | .301 | -.09 | -.25 | .08 |
| “taken seriously” | Constant | .00 | 5.80 | <.001 | .00 | .00 | .00 |
|  | Teaching condition | .07 | -.12 | .902 | -.01 | -.15 | .13 |
|  | Center 2 | .15 | 1.20 | .231 | .18 | -.12 | .48 |
|  | Center 3 | .09 | -.72 | .474 | -.06 | -.23 | .11 |
|  | Course focus | .13 | -.16 | .873 | -.02 | -.28 | .24 |
|  | Study year | .11 | -1.62 | .106 | -.17 | -.38 | .04 |
|  | Self-reported diagnostic knowledge | .08 | -1.84 | .067 | -.15 | -.32 | .01 |
|  | Knowledge test (t1) | .08 | .74 | .460 | .06 | -.10 | .22 |

|  |  | *SE* | *t value* | *p* | *β* | *95% CI for β* | |
| --- | --- | --- | --- | --- | --- | --- | --- |
|  |  |  |  |  |  | *lower* | *upper* |
| “positive relationship” | Constant | .00 | 5.21 | <.001 | .00 | .00 | .00 |
|  | Teaching condition | .07 | -1.42 | .156 | -.10 | -.24 | .04 |
|  | Center 2 | .15 | -1.66 | .098 | -.25 | -.54 | .05 |
|  | Center 3 | .09 | -2.54 | .012 | -.22 | -.39 | -.05 |
|  | Course focus | .13 | -.18 | .856 | -.02 | -.28 | .23 |
|  | Study year | .11 | 2.09 | .038 | .22 | .01 | .43 |
|  | Self-reported diagnostic knowledge | .08 | -1.78 | .076 | -.15 | -.31 | .02 |
|  | Knowledge test (t1) | .08 | -.22 | .827 | -.02 | -.18 | .14 |
| “not report everything” | Constant | .00 | 3.26 | .001 | .00 | .00 | .00 |
|  | Teaching condition | .07 | .52 | .603 | .04 | -.10 | .18 |
|  | Center 2 | .15 | 1.00 | .320 | .15 | -.15 | .45 |
|  | Center 3 | .09 | 1.24 | .218 | .11 | -.06 | .28 |
|  | Course focus | .13 | 1.67 | .097 | .22 | -.04 | .48 |
|  | Study year | .11 | .24 | .809 | .03 | -.18 | .24 |
|  | Self-reported diagnostic knowledge | .08 | -.12 | .904 | -.01 | -.17 | .15 |
|  | Knowledge test (t1) | .08 | -1.62 | .107 | -.13 | -.29 | .03 |
| “better understanding” | Constant | .00 | 3.31 | .001 | .00 | .00 | .00 |
|  | Teaching condition | .07 | 1.04 | .298 | .07 | -.07 | .21 |
|  | Center 2 | .15 | -1.78 | .077 | -.27 | -.56 | .03 |
|  | Center 3 | .09 | -1.78 | .076 | -.15 | -.32 | .02 |
|  | Course focus | .13 | -1.06 | .291 | -.14 | -.40 | .12 |
|  | Study year | .11 | -.44 | .662 | -.05 | -.26 | .16 |
|  | Self-reported diagnostic knowledge | .08 | 1.21 | .229 | .10 | -.06 | .26 |
|  | Knowledge test (t1) | .08 | 1.38 | .170 | .11 | -.05 | .27 |

|  |  | *SE* | *t value* | *p* | *β* | *95% CI for β* | |
| --- | --- | --- | --- | --- | --- | --- | --- |
|  |  |  |  |  |  | *lower* | *upper* |
| “enough detail” | Constant | .00 | 5.09 | <.001 | .00 | .00 | .00 |
|  | Teaching condition | .07 | -.69 | .488 | -.05 | -.19 | .09 |
|  | Center 2 | .15 | -.87 | .387 | -.13 | -.43 | .17 |
|  | Center 3 | .09 | -1.49 | .139 | -.13 | -.30 | .04 |
|  | Course focus | .13 | -1.17 | .244 | -.15 | -.41 | .11 |
|  | Study year | .11 | .38 | .707 | .04 | -.17 | .25 |
|  | Self-reported diagnostic knowledge | .08 | -1.28 | .202 | -.11 | -.27 | .06 |
|  | Knowledge test (t1) | .08 | 1.97 | .051 | .16 | -.001 | .32 |
| “helpfull” | Constant | .00 | 4.54 | <.001 | .00 | .00 | .00 |
|  | Teaching condition | .07 | -.37 | .715 | -.03 | -.17 | .12 |
|  | Center 2 | .15 | -1.17 | .245 | -.18 | -.48 | .12 |
|  | Center 3 | .09 | -.86 | .389 | -.08 | -.25 | .10 |
|  | Course focus | .13 | -.74 | .463 | -.10 | -.36 | .17 |
|  | Study year | .11 | .66 | .507 | .07 | -.14 | .29 |
|  | Self-reported diagnostic knowledge | .08 | -.72 | .474 | -.06 | -.23 | .11 |
|  | Knowledge test (t1) | .08 | 1.39 | .165 | .12 | -.05 | .28 |
